# Normalization of the right heart and the preoperative factors that influence the emergence PAH after surgical closure of atrial septal defect

**DOI:** 10.1186/s13019-020-01148-5

**Published:** 2020-05-20

**Authors:** Supomo Supomo, Agung Widhinugroho, Aditya Agam Nugraha

**Affiliations:** 1 Department of Surgery, Thoracic, Cardiac and Vascular Surgery, Faculty of Medicine, Public Health and Nursing, Universitas Gadjah Mada, Kesehatan Street No. 1, Yogyakarta, 55281 Indonesia; 2 Department of Surgery, General Surgery, Faculty of Medicine, Public Health and Nursing, Universitas Gadjah Mada, Yogyakarta, Indonesia; 3 Faculty of Medicine, Public Health and Nursing, Universitas Gadjah Mada, Yogyakarta, Indonesia

**Keywords:** Atrial Septal defect, ASD, Surgical closure, ASD closure, Normalization

## Abstract

**Background:**

Surgical closure of atrial septal defect (ASD) is contraindicated in the condition with severe pulmonary arterial hypertension (PAH), whereas ASD closure in an effective intervention to normalize the structure and function of the right heart after previously experiencing volume overload due to shunting from the defect. This study aimed to evaluate normalization of the right heart and emergence of PAH after surgical closure of ASD.

**Methods:**

This retrospective study was carried out in 45 patients over 18 years who had undergone surgical closure of ASD. The study has the aim to evaluate the morphological and functional parameters before and after the surgical approach and the preoperative factors that influenced the development of pulmonary arterial hypertension (PAP) after the ASD closure.

**Results:**

The majority of subjects were female (73.3%) although there were no significant differences between males and females from the various parameters. The average of mPAP in the group that experienced PAH was higher than non-PAH group after ASD closure (*p* = 0.019, 31.23 ± 12.70 mmHg vs 24.07 ± 13.08 mmHg). Significant differences were found in the Right Atrium (RA) dimension, Right Ventricle (RV) dimension, Tricuspid Regurgitation Velocity (TRV) and Tricuspid Annular Plane Systolic Excursion (TAPSE) between before and at 6 months after ASD closure (*p* = 0.000, *p* = 0.000, *p* = 0.000, *p* = 000, respectively). The sensitivity of the predictive model to estimate PAH at 6 months after surgical closure of ASD was 58%, with a specificity of 62.5%.

**Conclusion:**

Structural and functional normalization of the right heart occurs at 6 months after surgical closure of ASD with the decrease of RA and RV dimensions and improvement from tricuspid regurgitation. Emergence of PAH after ASD closure was influenced by higher mPAP before surgical approach.

## Background

Atrial Septal Defect (ASD) can go undiagnosed until adulthood [1, 2], and is one of the most common congenital heart diseases in adults [3]. Defect of the atrium often leads to volume overload in the right heart coupled with the potential risk of right heart failure, pulmonary arterial hypertension (PAH) and atrial arrhythmia [1, 4].. ASD closure is the current best treatment for ASD patients with stable hemodynamics [1]. ASD closure is usually done at infancy while still asymptomatic to prevent complications from shunt flow [3–5]. Early surgical closure of ASD has a good long-term outcome [2].

Pulmonary arterial (PA) pressure improvement can occur after ASD closure [6]. However, severe PAH can worsen the condition of the patients undergoing ASD closure because it requires partial shunting for blood flow to reduce right heart pressure [3]. Accordingly, severe PAH is contraindicated for surgical closure of ASD because of the risk of persistent PAH after ASD closure [7].

ASD is often followed by right heart enlargement due to volume overload [8]. Evidence of right heart enlargement from Transthoracic Echocardiography (TTE) can be used as an indication of ASD closure [4, 9]. Volume overload of the right atrium (RA) and right ventricle (RV) is known to be a consequence of the condition of untreated ASD, and the presence of persistent shunting can have an effect on the appearance of cardiac arrythmias [10].

This study aimed to evaluate the normalization of the right heart and preoperative factors that influence the development of PAH after ASD closure.

## Methods

This retrospective study was carried out in 45 patients over 18 years who had undergone surgical closure of ASD. The study has the aim to evaluate the morphological and functional parameters before and after the surgical approach and the preoperative factors that influenced the development of pulmonary arterial hypertension (PAP) after the ASD closure. Preoperative factors seen from the morphology and functional parameters before ASD closure by TTE examination were compared between PAH and non-PAH group at 6 months after ASD closure.

Data retrospectively taken from the Medical Record Installation of Dr. Sardjito General Hospital included: age, sex, body mass index (BMI), mean pulmonary arterial pressure (mPAP), left atrium (LA) dimension, diameter of ASD, right atrium (RA) dimension, right ventricle (RV) dimension, right atrium (RA) pressure, tricuspid regurgitation velocity (TRV), tricuspid annular plain systolic excursion (TAPSE), and ejection fraction (EF) from the results of TTE by cardiologists before surgery and 6 months after surgical closure of ASD. The mPAP prediction was mPAP obtained using predictive models to estimate mPAP after ASD closure by Supomo [7], and mPAP prediction = (0.24 x age) + (0.06 x mPAP before surgery) + (0.17 x RA dimension) + (0.47 x RV dimension) – 13.79. Pulmonary arterial hypertension (PAH) was defined as mean arterial pressure (mPAP) above 25 mmHg [2].

The Medical and Health Research Ethics Committee (MHREC) of the Faculty of Medicine, Public Health and Nursing, Universitas Gadjah Mada / Dr. Sardjito General Hospital, Yogyakarta, Indonesia (KE/1240/10/2019) approved this study. Informed consent was obtained from each patient before surgical closure of ASD.

Statistical analysis on all data was performed using SPSS version 23 (IBM Corp., Chicago). Continuous variables were in the form of mean ± standard deviation, while continuous variables with non-parametric data were presented in the median. Characteristic data between males and females were compared using student’s t-test to determine any significant difference between the variables as shown in Table 1, whereas non-parametric variables used the Mann-Whitney test. Evaluation of the independent variables that affect the development of PAH was done by comparing the preoperative condition of the PAH and non-PAH group before and at 6 months after ASD closure used the Mann Whitney test as shown in Table 2. PAH at 6 months after ASD closure was classified as mPAP > 25 mmHg based on the TTE examination at 6 months after surgical approach. The analysis results were considered to be significantly different if *p* < 0.05. Comparison of preoperative and 6 months postoperative data was performed using paired student’s t-test and for non-parametric data using the Wilcoxon test (Table 3). Predicted mPAP using Supomo [7] predictive model was compared with postoperative mPAP from TTE using unpaired student’s t-test, while calculating the sensitivity and specificity of formula compared with the gold standard of mPAP from TTE.

**Table 1:** Characteristics of data between male and female before surgery (*N* = 45 subjects)

Variables	Male (***n*** = 12)	Female (***n*** = 33)	***p***
Age (years)	33.45 ± 9.89	34.68 ± 9.90	0.753
BMI (kg/m^2^)	18.09 ± 2.98	19.60 ± 4.21	0.239
LA Dimension (mm)	35.81 ± 7.93	33.03 ± 7.58	0.425
RA Dimension (mm)*	47 (43–64)	47 (41–65)	0.198
RV Dimension (mm)	48.81 ± 9.43	43.50 ± 7.24	0.062
EF (%)	68.36 ± 8.27	69.24 ± 7.64	0.326
mPAP (mmHg)	28.76 ± 16.98	28.62 ± 11.66	0.985
RA Pressure (mmHg)*	8 (3–15)	8 (3–15)	0.809
TRV (m/s)	2.91 ± 0.89	3.07 ± 0.94	0.598
TAPSE (mm)	25.72 ± 6.46	27.71 ± 5.14	0.144
PAH	7 (58.3)	23 (69.7)	0.496

*=non parametric; BMI = Body Mass Index; mPAP = Mean Pulmonary Arterial Pressure; EF = Ejection Fraction; RA = Right Atrium; RV = Right Ventricle; TRV = Tricuspid Regurgitation Velocity; TAPSE = Tricuspid Annular Plane Systolic Excursion; LA = Left Atrium; PAH = Pulmonary Arterial Hypertension

**Table 2:** Comparison of preoperative factors in pulmonary arterial hypertension group and non-pulmonary arterial hypertension group before and at 6 months after ASD closure

Preoperative factors	Before Surgery		***p***	6 Months After Surgical Closure		***p***
	Pulmonary Hypertension (***N*** = 30)	Non-PAH (***N*** = 15)		Pulmonary Hypertension (***N*** = 29)	Non-PAH (***N*** = 16)	
Age (years)	36.12 ± 11.11	31.14 ± 5.91	0.049	35.32 ± 10.37	32.57 ± 8.74	0.144
BMI (kg/m2)	19.67 ± 4.25	18.28 ± 3.20	0.194	19.27 ± 3.83	19.00 ± 4.23	0.621
EF (%)	68.95 ± 8.74	69.07 ± 5.78	0.804	69.19 ± 8.54	68.64 ± 6.29	0.946
TRV (m/s)*	3.28 (0.53–4.30)	2.74 (1.47–3.89)	0.057	3.27 (0.53–4.30)	3.00 (0.59–4.30)	0.551
TAPSE (mm)	27.36 ± 6.05	26.78 ± 4.64	0.682	27.40 ± 5.82	26.71 ± 5.15	0.994
RA Dimension (mm)	50.04 ± 6.49	47.35 ± 6.03	0.180	49.32 ± 6.73	48.64 ± 5.91	0.640
RV Dimension (mm)	45.80 ± 7.38	43.57 ± 9.52	0.109	45.44 ± 7.42	44.21 ± 9.58	0.379
mPAP (mmHg)	35.84 ± 10.40	15.84 ± 5.32	0.000**	31.23 ± 12.70	24.07 ± 13.08	0.019**
RA Pressure (mmHg)*	8 (3–15)	8 (3–8)	0.549	8 (3–15)	8 (3–8)	0.916
LA dimension (mm)	34.12 ± 9.19	33.28 ± 4.03	0.440	34.92 ± 8.37	31.85 ± 6.06	0.171
Size of ASD (mm)	26.52 ± 8.47	27.14 ± 10.48	0.941	28.88 ± 7.73	22.93 ± 10.38	0.050

* = non parametric; ** = Significant difference; BMI = Body Mass Index; mPAP = Mean Pulmonary Arterial Pressure; EF = Ejection Fraction; RA = Right Atrium; RV = Right Ventricle; TRV = Tricuspid Regurgitation Velocity; TAPSE = Tricuspid Annular Plane Systolic Excursion; LA = Left Atrium; ASD = Atrial Septal Defect

**Table 3:** Comparison of the variables before and after ASD closure

Variables	Before	After	***p***	95%CI	Mean Differences
RA Dimension (mm)	48.84 ± 6.23	36.96 ± 6.03	0.000**	9.77–13.99	11.87 ± 7.01
RV Dimension (mm)	44.89 ± 8.11	34.80 ± 5.50	0.000**	7.71–12.46	10.09 ± 7.91
LA Dimension (mm)	34.13 ± 7.26	32.75 ± 6.75	0.184	−0.677-3.43	1.37 ± 6.84
TRV (m/s)	2.95 ± 0.98	2.23 ± 0.60	0.000**	0.40–1.04	0.72 ± 0.89
TAPSE (mm)	27.13 ± 5.42	14.46 ± 4.39	0.000**	10.69–14.64	12.66 ± 6.57
mPAP (mmHg)*	31.52 (5.65–52.45)	27.20 (2.50–51.00)	0.101		
RA Pressure (mmHg)*	8.00 (3.00–15.00)	3.00 (3.00–15.00)	0.403		
EF (%) *	69.50 (49.00–85.00)	69.00 (56.00–79.00)	0.404		

* = non parametric; ** = Significant Difference; mPAP = Mean Pulmonary Arterial Pressure; EF = Ejection Fraction; RA = Right Atrium; RV = Right Ventricle; TRV = Tricuspid Regurgitation Velocity; TAPSE = Tricuspid Annular Plane Systolic Excursion; LA = Left Atrium

## Results

The baseline characteristics of a total of 45 patients before ASD closure are shown in Table 1 by comparing between males and females. No significant difference in the patients’ condition before ASD closure was found between males and females, while the majority of patients were female with as many as 73.3% of the total subjects.

The analyses of the preoperative parameters in PAH and non-PAH group before and at 6 months after surgery are shown in Table 2. There were 30 patients with PAH before surgery, and 29 patients at 6 months after surgery who experienced PAH. The average age of the patients who experienced PAH before and at 6 months after surgery were 36.12 ± 11.11 years and 35.32 ± 10.37 years, respectively. The average preoperative mPAP was higher in PAH (35.84 ± 10.40 mmHg) than non-PAH (15.84 ± 5.32 mmHg) before ASD closure (*p* = 0.000), while at 6 months after surgery, the preoperative mPAP in PAH and non-PAH group were 31.23 ± 12.70 mmHg and 24.07 ± 13.08 mmHg (*p* = 0.019), respectively. Higher mPAP before ASD closure was found in PAH group at 6 months after ASD closure. Whereas BMI, EF, TRV, RA dimension, LA dimension, RV dimension, RA pressure and TAPSE did not show any significant differences between PAH and non-PAH group before and at 6 months after ASD closure.

Bivariate analyses of RA dimension, RV dimension, LA dimension, RA pressure, TRV, TAPSE, EF and mPAP before and at 6 months after ASD closure are shown in Table 3. There were significant differences in RA dimension, RV dimension, TRV and TAPSE before and at 6 months after ASD closure (*p* = 0.000, *p* = 0.000, *p* = 0.000, *p* = 000, respectively). Meanwhile, other variables such as LA dimension, RA pressure, mPAP and EF did not show any significant difference between before and at 6 months after ASD closure.

Comparison between estimated mPAP using the Supomo [7] predictive models and mPAP from TTE examination did not show any significant difference (*p* = 0.105) and used a cut off of 25 mmHg in PA pressure to categorize the patient’s condition as PAH. The diagnostic test to assess PAH at 6 months after ASD closure showed that the sensitivity of the Supomo [7] prediction model was 56% with a specificity of 62.5%.

## Discussion

The current gold standard to assess PA pressure to diagnose PAH is using the right heart catheterization (RHC), but TTE is more often used to diagnose PAH because of its availability [11]. This study used TTE as a tool to access the condition of the heart in ASD patients before surgery and at 6 months after surgical closure of ASD. PAH in congenital heart disease can occur due to progressive vascular remodelling [11]. Defect closure in ASD is indicated in the left to right shunt with the evidence of the right heart enlargement because of volume overload [9, 12, 13]. The closure of ASD causes protection of the right heart from volume overload and can also reduce the pulmonary arterial pressure [3]. In our study, most of the patients had an enlarged right heart as indicated by RA and RV dilatation from TTE examination. However, ASD closure is contraindicated in high pulmonary vascular resistant (PVR) conditions or in patients with pulmonary arterial pressure more than two-thirds of systemic arterial pressure [9, 12].

Surgical closure of ASD is an effective and safe method to correct the defect, with RV volume overload characterized by RA and RV dilatation as the most common criteria used as indication for ASD closure [13]. In our study, there were significant differences in RA and RV diameters before and at 6 months after surgical closure of ASD. There was a decrease in diameter of about 10 mm in both RA and RV diameters. Interventions in the form of closure of the ASD, including both surgical and transcatheter, have good efficacy in the normalization of the right heart chamber [2–5, 14]. Previous studies showed that there was a significant decrease in both horizontal and vertical dimensions of the RA before and after ASD closure [2, 3, 15]. In addition to the normalization of the right heart chamber, there is also a functional improvement of the right heart that can be seen from the improvement of TAPSE parameters in TTE after ASD closure, and this improvement can reduce pulmonary arterial pressure that was elevated before the closure [3, 15].

In our study, right heart function described by TAPSE and TR velocity had significant differences between before and at 6 months after ASD closure. TAPSE is the standard parameter for evaluating right ventricular function quantitatively [15]. In our study, we found a decrease in TAPSE at 6 months after surgical closure of ASD with an average reduction of 12.66 ± 6.57 mm. This result is similar to the findings of Akula et al. [15] which showed a decrease in TAPSE after ASD closure. This decrease in TAPSE indicates an improvement in the function of the right ventricle [15]. RV will accommodate smaller volumes after ASD closure due to the decreased dimension of RV, and this has an effect on improving stroke volume from the RV to pulmonary artery [14, 15].

Tricuspid regurgitation (TR) often occurs in adult ASD patients because of the shunt caused by ASD that is a consequence of right ventricle volume overload [16, 17] Our study showed similar results that all adult ASD patients experienced tricuspid regurgitation as seen from the TTE examination. Improvement from regurgitation state of tricuspid valve in long-term follow-up occurs after the closure of ASD, because of reverse remodelling that occurs after ASD closure [16–18]. The condition of regurgitation of the heart valve that includes the tricuspid valve will increase cardiovascular morbidity, reduce functional capacity, and increases the risk of heart failure and even death [16].

ASD closure is contraindicated in severe PAH, but there is currently no PA pressure and pulmonary vascular resistance index (PVRI) level set as a limit for ASD closure [7, 19]. Besides PAP, another parameter used is evidence of shunt left to right, with pulmonary vascular resistance (PVR) less than two-thirds of systemic vascular resistance (SVR) [19]. This study showed a slight decrease in pulmonary arterial pressure after ASD closure but the decrease was not statistically significant. Persistent elevated PAP can occur after ASD closure [16]. In ASD patients with an increase in PAP and Qp but who have normal PVR or only slightly increased, there is usually only minimal change in pulmonary vascular, so it is good to do surgical closure of ASD [20]. Conversely, complete change in pulmonary vascular can happen in the conditions with high increases in PAP and high PVR, so that the surgical closure of ASD is contraindicated [20].

In our study, the value of pulmonary artery pressure at 6 months after ASD closure was still above 25 mmHg. A predictive model of mean pulmonary arterial pressure by Supomo et al. [7] used several parameters from TTE examination before and at 6 months after surgical closure was retested in this study and showed no significant difference between mPAP from TTE and mPAP that was estimated with the predictive model (*p* = 0.105). The sensitivity of the diagnostic test for the development of pulmonary arterial hypertension at 6 months after ASD closure was 58% and the specificity was 62.5% by using a cut-off of 25 mmHg to define the patient’s condition as PAH [2]. Aging is a risk of PAH in ASD patients because it is a reflection of the duration of the shunt flow due to ASD [21]. The presence of shunt flow in ASD can cause an increase in pulmonary flow and has a prolonged effect on vascular hypertrophy and vasoconstriction in pulmonary arteries [21]. The right ventricle and atrial dimensions are related to the presence of PAH because in the PAH, the right ventricular and atrial dilatation often occur [2, 21]. Low sensitivity and specificity of the predictive model to predict PAH can be caused by PAH is a complex process that is not only influenced by the parameters examined in the previous study. Besides being influenced by the right heart dimensions, the emergence of PAH in ASD also influenced by the size of defect, respiratory disease, NYHA class III/IV, tricuspid regurgitation and the presence of arrhythmias [2, 6, 21], where these parameters have not been analyzed in previous studies. Persistent PAH can occur because of persistent elevated levels of PVR after ASD closure [2]. Although there is a decrease in PA pressure postoperatively, it rarely reaches normal values, especially in patients with advanced PAH before ASD closure [2].

One limitation of this study is that there was no randomization of subjects, because all ASD patients from 2017 to 2019 who underwent surgical closure of ASD were included in this study and the parameters used were only from TTE examination. Besides that, we had not included stratification of age, type and size of ASD, presence or absence of congenital heart disorder concomitant and the treatment that had been obtained in the analysis. Further study can use parameters that are the current gold standards such as using the right heart catheterization (RHC) to evaluate pulmonary arterial pressure and also need of complementary studies to evaluate the long-term evolution of PAH after the ASD closure.

## Conclusions

Surgical closure of ASD is an intervention that can normalize the anatomy and function of the right heart. Emergence of PAH after ASD closure was influenced by higher mPAP before surgical approach. Improvements to the atrial and ventricular dimensions occur at 6 months after ASD closure. Tricuspid regurgitation (TR), which often occurs in ASD, has significantly improved TR velocity at 6 months after ASD closure. Further study with more accurate parameters is needed to confirm the findings of our study.

## Data Availability

All data are included in the submission. The raw data are available from the corresponding author on reasonable request.

## Abbreviations

ASD: Atrial Septal Defect
PAH: Pulmonary Arterial Hypertension
mPAP: Mean Pulmonary Arterial Pressure
TTE: Transthoracic Echocardiography
RA: Right Atrium
RV: Right Ventricle
TRV: Tricuspid Regurgitation Velocity
TAPSE: Tricuspid Annular Plane Systolic Excursion
EF: Ejection Fraction
BMI: Body Mass Index
RHC: Right Heart Catheterization
PVR: Pulmonary Vascular Resistance
PVRI: Pulmonary Vascular Resistance Index
SVR: Systemic Vascular Resistance

## References

[1] Komar M, Przewlocki T, Olszowska M, Sobien B, Podolec P (2014). The benefit of atrial septal defect closure in elderly patients. Clin Interv Aging.

[2] Humenberger M, Rosenhek R, Gabriel H (2010). Benefit of atrial septal defect closure in adults: impact of age. Eur Heart J.

[3] Arafa OS, Aboelazm TH, Mostafa SA, Elemam AM, Abdelhakim A (2015). Transcatheter closure of atrial septal defect preserves right ventricular function. EC Cardiology.

[4] Kort HW, Balzer DT, Johnson MC (2001). Resolution of right heart enlargement after closure of secundum atrial septal defect with transcatheter technique. J Am Coll Cardiol.

[5] Castaldi B, Vida VL, Argiolas A (2015). Late electrical and mechanical remodeling after atrial septal defect closure in children: surgical versus percutaneous approach. Ann Thorac Surg.

[6] Balint OH, Samman A, Haberer K (2008). Outcomes in patients with pulmonary hypertension undergoing percutaneous atrial septal defect closure. Heart..

[7] Supomo S, Arjana AZ, Darmawan H (2018). Predictive model for secundum atrial septal defect closure with pulmonary artery hypertension in adult: when to close. Heart Surg Forum.

[8] Kumar P, Sarkar A, Kar SK (2019). Assessment of ventricular function in patients of atrial septal defect by strain imaging before and after correction. Ann Card Anaesth.

[9] Fraisse A, Latchman M, Sharma SR (2018). Atrial septal defect closure: indications and contra-indications. J Thorac Dis..

[10] Kucinska B, Werner B, Wróblewska-Kałużewska M (2010). Assessment of right atrial and right ventricular size in children after percutaneous closure of secundum atrial septal defect with Amplatzer septal occluder. Arch Med Sci.

[11] Nashat H, Montanaro C, Li W (2018). Atrial septal defects and pulmonary arterial hypertension. J Thorac Dis..

[12] Liava’a M, Kalfa D (2018). Surgical closure of atrial septal defects. J Thorac Dis..

[13] Rao P Syamasundar, Harris Andrea D (2017). Recent advances in managing septal defects: atrial septal defects. F1000Research.

[14] Balcı KG, Balcı MM (2015). Aksoy MM, et al remodeling process in right and left ventricle after percutaneous atrial septal defect closure in adult patients. Turk Kardiyol Dern Ars Arch Turk Society of Cardiology.

[15] Akula VS, Durgaprasad R, Velam V, Kasala L, Rodda M, Erathi HV (2016). Right ventricle before and after atrial septal defect device closure. Echocardiography..

[16] Takaya Y, Akagi T, Kijima Y, Nakagawa K, Ito H (2017). Functional tricuspid regurgitation after transcatheter closure of atrial septal defect in adult patients: long-term follow-up. JACC Cardiovasc Interv.

[17] Giamberti A, Chessa M, Ballotta A (2011). Functional tricuspid valve regurgitation in adults with congenital heart disease: an emerging problem. J Heart Valve Dis.

[18] Chen L, Shen J, Shan X, Wang F, Kan T (2018). Improvement of tricuspid regurgitation after transcatheter ASD closure in older patients. Herz..

[19] Jain S, Dalvi B (2018). Atrial septal defect with pulmonary hypertension: when/how can we consider closure?. J Thorac Dis.

[20] D'Alto M, Romeo E, Argiento P (2013). Hemodynamics of patients developing pulmonary arterial hypertension after shunt closure. Int J Cardiol.

[21] Yong G, Khairy P, De Guise P (2009). Pulmonary arterial hypertension in patients with transcatheter closure of secundum atrial septal defects: a longitudinal study. Circ Cardiovasc Interv.

